# The role of HIV as an independent risk factor to cervical HSIL recurrence

**DOI:** 10.61622/rbgo/2024rbgo85

**Published:** 2024-10-23

**Authors:** Fernanda Villar Fonseca, Newton Sérgio de Carvalho, Carlos Afonso Maestri, Manuella Fernandes Martins, Dora Pedroso Kowacs

**Affiliations:** 1 Universidade Positivo Curitiba PR Brazil Universidade Positivo, Curitiba, PR, Brazil.; 2 Universidade Federal do Paraná Curitiba PR Brazil Universidade Federal do Paraná, Curitiba, PR, Brazil.

**Keywords:** HIV infections, Uterine cervical neoplasms, Electrosurgery, Recurrence, Excision margins, Disease-free survival, Squamous intraepithelial lesions, Risk factors

## Abstract

**Objective:**

To evaluate the role of being human immunodeficiency virus (HIV) positive for predicting the risk of recurrence in women with a cervical high grade squamous intraepithelial lesion (HSIL) diagnosis.

**Methods:**

Retrospective observational case-control study, comprising HIV positive (case) and HIV negative (control) women in a 1:4 ratio. Women assisted by the Erasto Gaertner Hospital, between 2009-2018, with cervical HSIL diagnosis, submitted to treatment by Loop electrosurgical excision procedure (LEEP), and with a minimum follow-up of 18 months, were included. The immunological status, number and time to recurrence were analyzed, with p<0.05 considered significant. In a second analysis, only patients with free margins were evaluated.

**Results:**

The sample consisted of 320 women (64 cases and 256 controls). Presence of HIV, CD4 levels <200 and detectable viral load (CV) were associated with high risk of recurrence, with odds ratio (OR) of 5.4 (p<0.001/95CI:2.8-10); 3.6 (p<0.001 /IC95:0.6-21.1) and 1.8 (p=0.039 /IC95:0.3-9.3), respectively. In the sample with free margins (n=271), this risk was also higher among seropositive patients, with OR 4.18 (p=0.001/95CI:1.8-9.2).

**Conclusion:**

HIV is an independent risk factor for cervical HSIL recurrence and reduced disease-free survival time. Glandular involvement, compromised margins, undetectable CV and CD4<200 also increase the risk of relapse.

## Introduction

The appropriate use of antiretroviral therapy (ART) has significantly increased the survival of people living with HIV and also increased the prevalence of diseases associated with the virus, such as infections by other viruses/bacteria and the development of premalignant and/or cancerous lesions.^(1-3)^ Among the diseases associated with HIV in the female population, co-infection with human papillomavirus (HPV) stands out, with a higher risk of developing cervical intraepithelial neoplasia (CIN) and progression to cervical cancer (SCC).^(4)^

SCC is the most common malignancy in women with acquired immunodeficiency syndrome (AIDS), which is secondary to HIV infection, and the third most common type of cancer in Brazilian women, surpassed only by breast, colon, and rectal cancer.^(5,6)^ In the United States, its incidence is 66% higher in HIV-positive individuals than in those who are not infected with the virus.^(7)^

Cervical intraepithelial neoplasia, precursor lesions of SCC, can be low or high grade, with high grade (HSIL, CIN 2/3) considered pre-malignant, which can progress to cervical cancer. Among the risk factors described in the literature for this progression are: advanced age, nulliparity, high-grade injury, oncogenic HPV serotypes, persistent HPV infection, HIV co-infection, recurrent cervical neoplasia, compromised margin status, reduced CD4 level, glandular extension, and presence of some associated sexually transmitted infections (STIs).^(4,5,8-15)^

In this context, the purpose of this study is to identify the clinical and statistical significance of HIV seropositivity as an independent prognostic factor for predicting the recurrence of high-grade intraepithelial neoplasia (HSIL or CIN 2/3) treated with conization by LEEP technique, and its relationship with the use of antiretroviral therapy, the immune status of seropositive patients, through CD4 count and/or viral load, and disease-free time.

## Methods

A retrospective case-control study was conducted in a 1:4 ratio, including women seen between January 2009 and December 2018 at the Cervical Pathology Service of Erasto Gaertner Hospital (HEG) in Curitiba, Brazil, with a histological diagnosis of CIN 2 or 3, who were treated with conization surgery using LEEP technique and underwent post-treatment clinical follow-up for a minimum of 18 months to control for the risk of disease recurrence. A total of 320 women were included, with 64 HIV-positive (cases) and 256 HIV-negative (controls).

The patients were divided into two groups:

- GROUP 1 (CASES): Women who were HIV positive, diagnosed with cervical high-grade intraepithelial lesion, treated with conization by high-frequency surgery, with a follow-up period of at least 18 months, with or without recurrence of CIN.
- GROUP 2 (CONTROLS): Women who were HIV negative, diagnosed with cervical high-grade intraepithelial lesion, treated with conization by high-frequency surgery, with a follow-up period of at least 18 months, with or without recurrence of CIN.

The epidemiological, serological, and histopathological data from the surgical specimen were correlated with the patients’ progression and evaluated for their ability to predict the recurrence of neoplasia.

The inclusion criteria were women treated at the Cervical Pathology/Colposcopy Unit of HEG, who underwent cervical cytology, colposcopy, and biopsy confirming high-grade intraepithelial lesion (CIN 2/3); who were treated with conization surgery resulting in the identification of any grade of CIN in the histological specimen; with post-treatment clinical follow-up for a minimum of 18 months, through cervical cytology, colposcopy, and biopsy at six-month intervals, to identify cure and/or disease recurrence.

The exclusion criteria included medical records with incomplete clinical data, follow-up time post-conization of less than 18 months, patients who underwent hysterectomy for benign conditions, presence of invasive carcinoma in the biopsy or conization product and absence of evidence of cervical disease.

All analyzed patients had histological results of CIN 2 or 3 in the cervical biopsy before conization, and all were tested for the presence or absence of HIV in serology. Disease recurrence was determined by the presence of CIN 2 or 3 in histology during post-surgical clinical follow-up.

Data collection on HIV seropositivity, use of antiretroviral therapy, and CD4/viral load count was performed by analyzing data in the electronic medical record of each patient.

The HIV seropositivity test was performed, after the diagnosis of CIN 2-3, prior to the conization surgery, in all patients of the service whose serological status was unknown, and, before the execution, the test was authorized by the patients who signed the consent form in the hospital laboratory mentioned.

For each patient in the Case group (HIV seropositive), 4 patients in the Control group (HIV seronegative) were randomly selected, through matched selection among all treated patients for CIN 2-3 in the same period, obeying the maximum similarity between the groups, since the CASE and CONTROL patients have geographic area identity, similar mean age, and similar socio-economic and cultural factors within the served community.

The data were analyzed using IBM SPSS Version 19.0 with a confidence interval (CI) of greater than 95% and a significance level of 5% (p ≤ 0.05). Recurrence between groups was analyzed by the chi-square test and/or Fisher's exact test, and the p-value was identified and calculated as the OR for the purpose of calculating the risk ratio of HIV as an independent predictor for recurrence of CIN 2-3. A Kaplan-Meier survival curve was performed to analyze the correlation of recurrence rate in HIV-positive patients with time until recurrence in the two groups.

This study was approved by the Ethics and Research Committee of the Erasto Gaertner Hospital under the protocol number 3481171/ CAEE 14934719.1.0000.0098.

## Results

Three hundred and twenty women were included in the study (global sample, n=320), with 64 HIV-positive (case group) and 256 HIV-negative (control group). The mean age of the participants was 42±9.7 (case) and 40±10 (control), ranging from 21 to 74 years and 22 to 76 years, respectively. There was no significant difference between the groups regarding age, parity, and smoking. Condom use was more frequent among seropositive patients (44%) than seronegative ones (19.5%). Table 1 shows the epidemiological data of the global sample.

**Table 1:** Comparative Epidemiological Data of global sample: Cases x Controls

Data		Group				p value
		Case (n=64) (HIV positive)		Control (n=256) (HIV negative)		
		X+SD	CI95	X+SD	CI95%	
Age		42±9.7	39-44	40±10	39-41	
Parity		2.1±1.6	2.1-2.9	2.4±1.8	2.2-2.7	
		%		%		
Smoking habit	Yes	62		71		0.165
	No	38		29		
Condom use	Yes	44		19.5		
	No	56		80.5		

* values with statistical significance (p<0.05)

Regarding the histopathological findings and results related to the conization product, shown in table 2, a significant relationship was observed between the presence of HIV and the absence of glandular extension (p=0.011), and the presence of compromised margins in the conization (p=0.001).

**Table 2:** Comparative data related to the Conization Product: Cases x Controls

Data		Group				p value
		Case (n=64) (HIV positive)		Control (n=256) (HIV negative)		
		n(%)		n(%)		
Histological grade	CIN 2	43/64(67)		151/256(59)		0.255
	CIN 3	21/64(33)		105/256(41)		
Glandular extension	Present	27/64(42)		154/256(60)		0.011*
	Absent	37/64(58)		102/256(40)		
Conzation margins	Free	45/64(70)		226/256(88)		0.001*
	Compromised	19/64(30)		30/256(12)		
		**X+SD**	**CI95%**	**X+SD**	**CI95%**	
Endocervical canal lenght removed (cm)		2.7±0.89	2.5-2.9	2.7±0.76	2.6-2.8	
Cervix volume removed (cm^3^)		6.6±2.5	0.4-12.9	3.8±1.9	1.4-6.1	

* values with statistical significance (p<0.05)

Only 52 participants had a recurrence of HSIL after LEEP, with 25 of them being in the case group (HIV-positive), resulting in a recurrence rate of 39% (25/64) in the CASES versus 10% (27/256) in the CONTROLS (p<0.001). After univariate analysis, only the variables compromised margins and seropositivity for HIV were significant for lesion recurrence (Table 3). The frequency of recurrence was three times higher in the group of HIV-positive patients - case group (25/64-39%) than in the seronegative group - control group (27/256-10%). While the chance of CIN2+ recurrence in seropositive patients was 5 times higher than in seronegative patients (OR 5.4 / 95% CI: 2.8-10, p<0.001).

**Table 3:** Comparative data related to recurrence of HSIL treated by LEEP

Data		Recurrence (n=52)	No recurrence (n=268)	p value <0.05	OR	CI95%
		n (%)	n (%)			
Smoking habit	Yes	20/52(39)	78/268(29)	0.13	1.6	0.8-3
	No	32/52(61)	190/268(71)			
Histological grade	CIN 2	29/52(56)	165/268(62)	0.44	0.78	0.4-1.4
	CIN 3	23/52(44)	103/268(38)			
Glandular extension	Present	29/52(56)	152/268(57)	0.89	0.96	0.5-1.7
	Absent	23/52(44)	116/268(43)			
Conization margins	Free	30/268(11)	238/268(89)	<0.001*	4.5	2.3-9
	Compromised	19/52(36)	33/52(64)			
HIV	Yes	25/52(48)	39/268(15)	<0.001*	5.4	2.8-10
	(n=64)	25/64(39)	39/64(61)			
	No	27/52(52)	229/268(85)			
	(n=256)	27/256(10)	229/256(90)			

* values with statistical significance (p<0.05)

**Figure 1:**
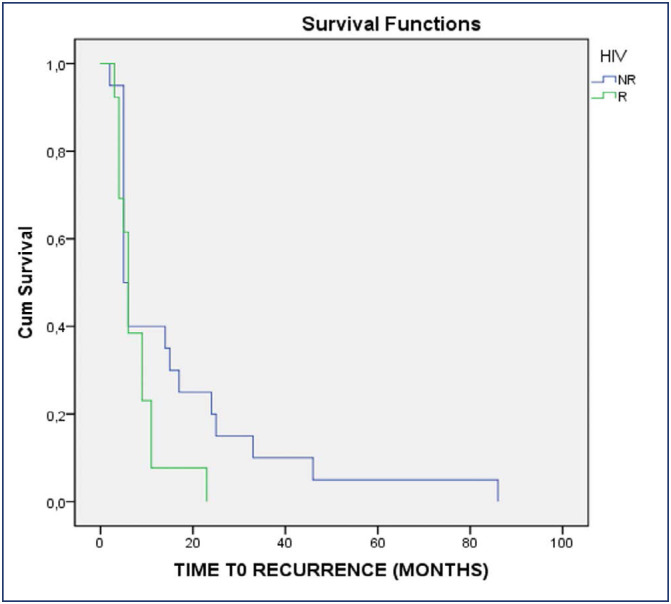
Kaplan Meir survival graph. / Comparative Analysis of disease-free time between HIV positive and HIV negative women, after HSIL treatment, and margin free conization, in months. Log Rank (Mantel-Cox): chi-Square 2,915 / p= 0.088

As shown in the Kaplan-Meier Curve (Figure 1), the disease-free survival time (average time to recurrence of NIC2+) among seropositive women (8±9 months /95% CI: 4-11/ min 2 and max 45 months) was almost half the time of seronegative women (13±18 months /95% CI: 6-20/ min 2 and max 86 months).

*NR - HIV negative (Not reagente for HIV) / R - HIV positive (reagente for HIV)

The presence of compromised margins in the conization specimen was the second most relevant factor for the risk of NIC 2+ recurrence (p<0.001/OR 4.5 - IC95: 2.3-9). Thus, with the aim of reducing methodological bias, a secondary analysis of the data was performed, excluding participants with compromised margins (n=49) and evaluating only those with free margins (n=271). In this new sample, 226 women were seropositive for HIV (case group) and 45 were seronegative (control group). In this secondary analysis, the rate of neoplasia recurrence was 29% (13/45) in seropositive patients and 9% (20/226) in seronegative patients, determining an OR of 4.18 (IC95: 1.8-9.2/p: 0.001), confirming the data from the global sample. Among the seropositive patients in the secondary sample, 9/28 were taking antiretroviral therapy, 52% had CD4>200, 80% had CD4<200, 4/7 had a detectable viral load (VL), and 3/7 had undetectable VL. The relationship between the immunological and viral status of seropositive patients in both the global and margin-free samples is shown in table 4. In the global sample, low CD4 levels, i.e. <200, (p<0.001/OR 3.6 IC95:0.6-21.1) and detectable VL (p=0.039/OR 1.8 - IC95:0.3-9.3) were significant variables for recurrence. However, when considering only the margin-free cases, only low CD4 levels (p=0.007/OR 2.3 - IC95:0.3-17) showed statistical significance.

**Table 4:** Recurrence data related to immunological status of HIV positive women in global sample and in margin free cases (n=271)

Global sample		Recurrence rate	p value	OR (odds ratio)
		n(%)	(n=320)	
ART in use				
	Yes	20/44(45)	p = 0.169	2.5 (IC95: 0.7-8)
	No	5/20(25)		
CD4 counts				
	>200	12/23(52)	p < 0.001*	3.6 (IC95: 0.6-21.1)
	<200	8/10(80)		
Viral load				
	Detectable	9/14(64)	p = 0.039*	1.8 (IC95: 0.3-9.3)
	Not detectable	5/10(50)		
**Margin free cases**			**(n=271)**	
ART in use				
	Yes	9/28(32)	p = 0.737	1.5 (IC95: 0.3-6)
	No	4/17(23)		
CD4 counts				
	>200	6/13(46)	p= 0.007*	2.3 (IC95: 0.3-17)
	<200	4/6(67)		
Viral load				
	Detectable	4/7(57)	p = 0.093	1.7 (IC95: 1.8-9.2)
	Not detectable	3/7(43)		

* The chance of recurrence (OR) was calculated in relation to: ART in use, CD4 <200 and Detectable Viral load; Unknown data regarding CD4, viral load and use of ART were excluded

## Discussion

Given that HPV and HIV have many similarities in terms of risk factors and entry route, coinfection is not uncommon. It is well known that HIV immunosuppression plays an important role in the establishment of HPV in the uterine cervix, resulting in a greater persistence of HPV, as well as a higher risk of progression to high-grade lesions, recurrence, and refractoriness to treatment in coinfected patients compared to seronegative individuals.^(16-21)^ A study conducted in São Paulo detected HPV DNA in 204 of the 208 HIV-seropositive women studied.^(19)^ Since the study considered the presence of viral DNA, the results included both patients who had normal and abnormal oncotic cytological results.

The present study confirmed that recurrence of CIN 2+ is three times higher in women with HIV, and is associated with glandular involvement and margin involvement, which is consistent with other studies such as the case-control study by Lodi et al.^(20)^ In their study, the same variables were associated with recurrence, with p-values of 0.001, 0.000, and 0.02, respectively. However, unlike our study, Lodi et al.^(20)^ considered the presence of both CIN 1 and CIN 2 as recurrence, and had a sample size of only 138 women, 70 seropositive and 68 seronegative.

The disease-free survival time represents the time it takes the patient to experience neoplasia recurrence. In this study, it was observed that HIV-positive women take half the time of HIV-negative women to relapse (8±9 months vs. 3±18 months, respectively), suggesting that HIV-positive patients have a shorter window for recurrence to occur. Minkoff et al.^(22)^ found that HIV-positive women were more likely than HIV-negative women to acquire new HPV infections and less likely to clear these infections.

Since margin involvement was highly associated with CIN2+ recurrence, a secondary sample of only patients with clear margins was analyzed to avoid bias in the research. Clear margins do not necessarily exclude lesion recurrence.^(16)^ Therefore, it was possible to observe in this group a higher recurrence in seropositive patients, seen in 29% (13/45), compared to seronegative patients, in 9% (20/226). A study obtained very similar results, where, when analyzing 94 HIV-positive and 107 HIV-negative patients with varying degrees of CIN, it was concluded that the recurrence rate for positive patients was 33% while for negative patients it was 8.4%.^(16)^

The main limitation of this study was the lack of data on the immunological status of the patients in the second sample. As a retrospective study, there was a gap in the data regarding CD4 lymphocyte counts and detectable or undetectable viral load of the patients. This influenced the analysis of the recurrence rate, in which only those on antiretroviral therapy had their information fully described in medical records. The recurrence rate of patients on ART was 45% (20/44), while those not on ART was 25% (5/20). However, data on CD4 counts were reported for 33 patients and viral load data for 24 patients. The correlation between recurrence rate and low CD4 lymphocyte counts suggests a strong link.^(16,22)^

It is possible to conclude that HIV seropositivity is closely related to higher prevalence of HPV infection, higher recurrence rates, and shorter disease-free survival time in women with high-grade intraepithelial neoplasia, even after undergoing conization treatment, as seen in previous studies. The low CD4 T-cell count (<200) also appears to be linked to the recurrence rate of the disease, as well as the use of ART.

## Conclusion

The presence of HIV infection is an independent risk factor for recurrence of high-grade cervical intraepithelial neoplasia after conization, with a shorter time interval to recurrence compared to the rest of the population. Glandular involvement, margin involvement, detectable viral load, and CD4 count <200 are also associated with a higher risk of recurrence.
